# Comparing sleep measures in cancer survivors: Self-reported sleep diary versus objective wearable sleep tracker

**DOI:** 10.21203/rs.3.rs-3407984/v1

**Published:** 2023-10-09

**Authors:** Xiaotong Li, Jun J. Mao, Sheila N. Garland, James Root, Susan Q. Li, Tim Ahles, Kevin T. Liou

**Affiliations:** Memorial Sloan Kettering Cancer Center; Memorial Sloan Kettering Cancer Center; Memorial University of Newfoundland, Newfoundland and Labrador; Memorial Sloan Kettering Cancer Center; Memorial Sloan Kettering Cancer Center; Memorial Sloan Kettering Cancer Center; Memorial Sloan Kettering Cancer Center

**Keywords:** Sleep tracker, Insomnia, Sleep diary, Cancer, Fitbit

## Abstract

**Purpose:**

Cancer survivors are increasingly using wearable fitness trackers, but it’s unclear if they match traditional self-reported sleep diaries. We aimed to compare sleep data from Fitbit and the Consensus Sleep Diary (CSD) in this group.

**Methods:**

We analyzed data from two randomized clinical trials, using both CSD and Fitbit to collect sleep outcomes: total sleep time (TST), wake time after sleep onset (WASO), number of awakenings (NWAK), time in bed (TIB) and sleep efficiency (SE). Insomnia severity was measured by Insomnia Severity Index (ISI). We used the Wilcoxon Singed Ranks Test, Spearman’s rank correlation coefficients, and the Mann-Whitney Test to compare sleep outcomes and assess their ability to distinguish insomnia severity levels between CSD and Fitbit data.

**Results:**

Among 62 participants, compared to CSD, Fitbit recorded longer TST by an average of 14.6 (SD = 84.9) minutes, longer WASO by an average of 28.7 (SD = 40.5) minutes, more NWAK by an average of 16.7 (SD = 6.6) times per night, and higher SE by an average of 7.1% (SD = 14.4); but shorter TIB by an average of 24.4 (SD = 71.5) minutes. All the differences were statistically significant (all p < 0.05), except for TST (p = 0.38). Moderate correlations were found for TST (r = 0.41, p = 0.001) and TIB (r = 0.44, p < 0.001). Compared to no/mild insomnia group, participants with clinical insomnia reported more NWAK (p = 0.009) and lower SE (p = 0.029) as measured by CSD, but Fitbit outcomes didn’t.

**Conclusions:**

TST was the only similar outcome between Fitbit and CSD. Our study highlights the advantages, disadvantages, and clinical utilization of sleep trackers in oncology.

## Introduction

Up to 50% of newly diagnosed or recently treated cancer patients experience insomnia symptoms, and up to 75% of survivors experience chronic insomnia post-treatment^1^. Poor sleep is significantly associated with multiple problems, such as severe fatigue, mental disorders, worse physical function, and lower overall quality of life in individuals with cancer ^2–4^. However, poor sleep is often under-reported by most cancer patients: They seldom discuss sleep with health care providers because they perceive it to be less important to their physicians compared to cancer, cancer treatments, or other comorbid symptoms such as pain^2,5^. As a result, sleep problems are often ignored or inadequately managed.

Sleep diaries are an essential aspect of insomnia assessment and treatment and are used to guide clinical recommendations ^6^. However, patients often find diaries to be laborious, accuracy can be questionable, and non-adherence is common ^7^. Sleep-tracking offers a potentially efficient way to help clinicians understand and address patients’ sleep patterns in a timely fashion, thereby reducing the possibility of developing severe or chronic insomnia and other comorbidities in the future resulting from inadequate management of sleep difficulties ^8–11^. Compared to traditional self-reported sleep measures, such as a sleep diary, wearable devices allow for the collection of continuous and dynamic sleep outcomes in real-time ^12^. Furthermore, they are user-friendly, decrease self-reporting bias, and reduce patients’ burden from completing and submitting questionnaires, particularly during severe illness or over long periods of time^13^. These wearable devices are becoming increasingly prevalent in both the cancer and general populations, particularly due to the strong demand for remote and self-monitoring health brought about by the outbreak of COVID-19 ^14^. In 2019, the global wearable medical device market size was USD 29.76 billion and is projected to reach USD 195.57 billion by 2027 ^15^. Studies have also shown that these devices, including Fitbit, exhibit high sensitivity in detecting sleep when compared to gold-standard sleep measurement polysomnography (PSG) ^16^.

However, in the oncology setting, wearable devices are most commonly used for physical activity assessment, and only a few studies have used them for sleep measurement ^14,17^. Currently, there is no evidence showing whether wearable sleep trackers are a comparable alternative to traditional self-reported sleep diaries in the cancer population. Understanding whether and how commercial wearable sleep trackers could be used is important for establishing convenient and accurate sleep measurements, guiding the proper use of these new technologies, reducing excessive medical care, and informing future research in cancer care.

Therefore, we aim to compare sleep outcomes from self-reported sleep diary and wearable sleep tracker (Fitbit), using baseline data from two clinical trials. Our findings may inform the proper use of sleep trackers in oncology care and research.

## Methods

### Study Design, Participants, and Procedures

The current study was conducted using baseline data from two randomized clinical trials that evaluated the effect of acupuncture on cognitive and/or sleep difficulties in cancer survivors. The first trial CLARITY (NCT04007770) was a 2-arm, parallel, pilot study that compared acupuncture against sham acupuncture (SA) among diverse cancer survivors with perceived cognitive difficulties; the second trial ENHANCE (NCT04837820) is an ongoing 3-arm parallel randomized controlled trial (RCT) that compares acupuncture against SA and wait list control among breast cancer survivors with both cancer-related cognitive difficulties (CRCD) and insomnia. Both studies were approved by the institutional review board at Memorial Sloan Kettering Cancer Center (IRB number, CLARITY: 19–179; ENHANCE: 20–124).

Both trials enrolled English-speaking adult cancer survivors who reported moderate or greater perceived CRCD (a score of “quite a bit” or “very much” on at least one of the two items that specifically assess concentration and memory problems on the EORTC QLQ-C30 [version 3.0]) ^18,19^. Participants also reported that their cognitive functions have worsened since their cancer diagnosis by replying “Yes” to all of the following questions: 1) Do you think or feel that your memory or mental ability has gotten worse since your cancer diagnosis? 2)Do you think your mind isn’t as sharp now as it was before your cancer diagnosis? 3) Do you feel like these problems have made it harder to function on your job or take care of things around the home? Active cancer treatment (surgery, chemotherapy, and/or radiation therapy) was completed at least one month prior to study enrollment. Patients receiving maintenance cancer treatment with hormonal or targeted therapies were eligible provided that dose had been stable for the past 4 weeks and no plans to initiate or change hormonal or targeted therapy in the coming 8 weeks. Patients were not eligible if diagnosed with Alzheimer’s disease, vascular dementia, Parkinson disease, or other organic brain disorders, if they had scores of ≥ 10 on the Blessed Orientation-Memory-Concentration (BOMC) screening instrument, if they had primary psychiatric disorders not in remission, or if they altered dose of somnogenic medications (e.g., hypnotics, sedatives, antidepressants) in past 8 weeks.

In addition to the eligibility criteria above, participants in the ENHANCE study were required to score eight or greater on the Insomnia Severity Index (ISI). The ENHANCE study enrolled stage 0 to III breast cancer survivors while CLARITY also enrolled survivors with other cancer types (colorectal, prostate, or gynecological cancer).

All eligible participants provided informed consent and completed baseline assessments, including demographic characteristics (age, gender, race, employment, and education), clinical factors (cancer type, cancer stage, cancer treatment, and years since cancer diagnosis), and sleep measurements (Fitbit, ISI, and sleep diary). This secondary analysis only included data collected at baseline.

### Outcomes

#### Insomnia Severity Index

Self-reported insomnia severity was measured by ISI, which has been validated in the cancer population and is widely used for evaluating perceived insomnia symptom severity^20,21^. The ISI includes 7 items for assessing the severity of both nighttime and daytime components of insomnia over the prior 2 weeks^22^. Each item is rated on a 5-point Likert scale, with a higher score indicating more severe insomnia severity. The total score ranges from 0 to 28. Clinical meaningful cutoff values for the ISI are as follows: <8 (no insomnia), 8 to 14 (mild insomnia), 15 to 21 (moderate insomnia), and 22 to 28 (severe insomnia).

#### Consensus Sleep Diary

The Consensus Sleep Diary (CSD) is a standardized instrument used to track self-reported nightly sleep patterns and quality ^23^. We used participants’ CSD reports to calculate sleep efficiency (SE), sleep onset latency, wake after sleep onset (WASO), total sleep time (TST), time in bed (TIB), number of awakenings (NWAK), sleep quality, and terminal wakefulness. Every participant was asked to record their sleep condition every morning for one week at baseline, which was also the week when they wore the Fitbit. We also used the CSD to provide additional context for interpreting the Fitbit data, helping to distinguish daytime naps from nocturnal sleep. Since available Fitbit data was limited to TST, WASO, TIB, NWAK, and SE, we only examined these specific CSD outcomes in the analyses.

#### Wearable device Fitbit

The Fitbit Charge 4 was used to measure sleep by using both accelerometry and heart rate data, which were analyzed using proprietary algorithms to identify sleep-wake activity^24,25^. We used a research iPad to set up sleep tracker emails for each patient, paired with their own account. Data from the CLARITY trial was uploaded to a research computer, and data from the ENHANCE trial was transferred directly from the Fitbit Cloud to a remote monitoring dashboard created by MSK IT department to enhance data safety. Participants were instructed to wear the Fitbit on their wrist continuously for a whole week at baseline. We collected the same sleep outcomes as CSD for analysis.

#### Definition of sleep outcomes measured by CSD and Fitbit

TST is the total amount of time that people spend sleeping, from the beginning of the sleep episode to the end, excluding any awake time in between. When using sleep diaries, this is typically calculated from other self-report variables (TIB-SOL-WASO). WASO is the total amount of time awake that occurs after initial sleep onset and before terminating the sleep period (excluding SOL). NWAK refers to the total number of times that a person wakes up during the entire sleep episode. TIB refers to the total amount of time in bed, starting from the moment of intention to fall asleep and concluding with the final arising. SE is the percent of time in bed spent asleep and is calculated by dividing TST by TIB and multiplying the result by 100 to convert it to a percentage.

#### Statistical analysis

Descriptive statistics were used to assess sleep outcomes measured by Fitbit and CSD at baseline, as well as demographic and clinical characteristics (e.g., age, gender, and cancer type). We used Wilcoxon Signed Ranks Test to compare whether Fitbit and CSD provided significantly different sleep metrics. The Spearman’s rank correlation coefficient was used to evaluate the association between Fitbit and CSD on all sleep outcomes. The correlation coefficients range from − 1 to + 1, with 0 indicating no correlation and 1 or −1 indicating a complete positive or negative correlation, respectively. The cut points for interpreting Spearman’s rank correlation coefficient were 0.0 to 0.2 (or 0.0 to − 0.2) indicating a very weak or no correlation; 0.2 to 0.4 (or − 0.2 to − 0.4) indicating a weak correlation; 0.4 to 0.6 (or − 0.4 to − 0.6) indicating a moderate correlation; 0.6 to 0.8 (or − 0.6 to − 0.8) indicating a strong correlation; and 0.8 to 1.0 (or − 0.8 to − 1.0) indicating a very strong correlation ^26,27^. For the ISI assessment, we dichotomized participants who reported “0–8” and “9–14” as “no/mild insomnia” and those reported “15–21” and “22–28” as “clinical insomnia”. Then, we used the Mann-Whitney Test to compare the sleep outcomes measured by Fitbit and CSD between patients with no/mild insomnia and those with clinical insomnia as assessed by ISI. All analyses were two-sided, and a p-value of less than .05 was considered statistically significant. We conducted all statistical analyses using SPSS (version 26; IBM Corp).

## Results

### Patients Characteristics

Table 1 shows participants’ baseline characteristics (N = 62). The mean age was 57.8 years (standard deviation [SD], 11.7). Most participants were women (56, 90.3%) and white (42, 67.7%), and 53 (86.9%) had an education level of college or higher. Almost half (30, 48.4%) had a full-time job. The most common cancer type was breast (44, 71.0%) and the most common cancer stage was 0-I (30, 48.4%). The mean time since cancer diagnosis was 5.9 years (SD, 7.7). The mean ISI score was 14.5 (SD = 5.7).

### Self-reported versus wearable tracker sleep outcomes

Table 2 shows sleep metrics, including TST, WASO, NWAK, TIB, and SE, measured by both Fitbit and CSD. Compared to CSD, Fitbit reported longer TST by an average of 14.6 (SD = 84.7) minutes, longer WASO by an average of 28.7 (SD = 40.5) minutes, more NWAK by an average of 16.7 (SD = 6.6) times, and higher SE by an average of 7.1% (SD = 14.4), and shorter TIB by an average of 24.4 (SD = 71.5) minutes. Additionally, Fitbit significantly differed from CSD in all sleep outcomes, including WASO, NWAK, TIB, and SE (all *p* < 0.05), except for TST (*p* = 0.38). Notably, the NWAK reported by Fitbit was approximately 7 times more than that reported by CSD, on average.

**Figure 1:**
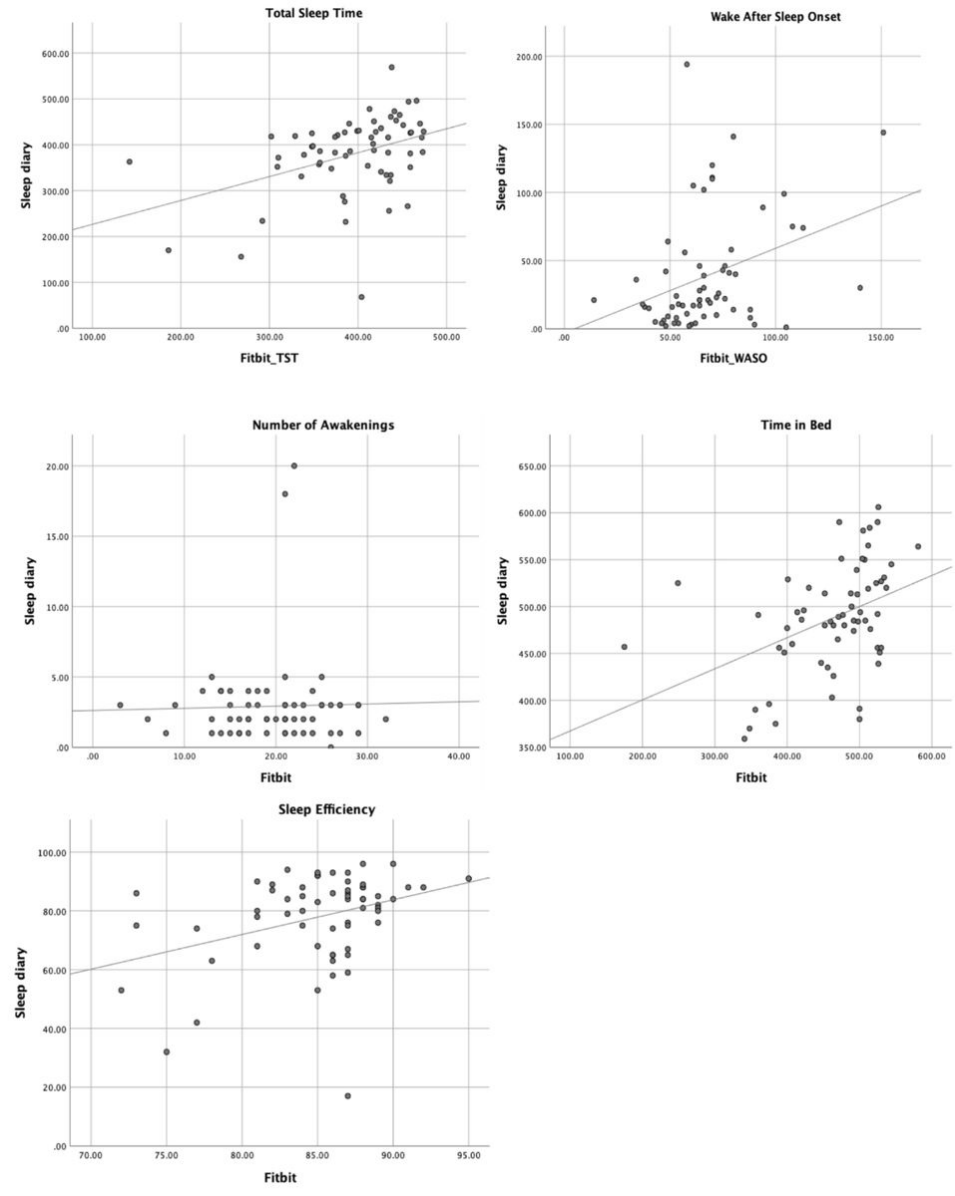
Correlation between Fitbit and CSD

Spearman’s rank correlation coefficients show the correlation between Fitbit and CSD on all sleep outcomes for all 62 participants (Fig. 1). These results indicate moderate correlations on TST (*r* = 0.41, *p* = 0.001) and TIB (*r* = 0.44, *p* < 0.001) and weak correlations on WASO (*r* = 0.36, *p* = 0.004) and SE (*r* = 0.29, *p* = 0.022). There was no significant correlation (*r*=−0.04) between Fitbit and CSD on the NWAK (*p* = 0.75).

Table 3 shows that sleep outcomes assessed by Fitbit did not differ significantly between participants with no/mild insomnia and those with clinical insomnia (all *p* > 0.05). However, when sleep outcomes were measured by CSD, both NWAK and SE showed statistically significant differences between these two populations: participants with clinical insomnia reported 1.8 times more NWAK (*p* = 0.009) and 6.9% lower SE (*p* = 0.029) than those with no/mild insomnia.

## Discussion

To our knowledge, this is the first study to compare sleep outcomes obtained from Fitbit versus CSD in cancer survivors. Our findings suggest that Fitbit may be comparable to CSD when assessing TST but may not be a suitable alternative to CSD for other sleep outcomes in cancer survivors. The choice of optimal sleep measure requires further research and may ultimately depend on their feasibility and practical considerations, as well as the goals of research and clinical applications.

In recent years, an increasing number of studies have used commercial wearables to measure sleep in clinical trials among cancer patients ^28,29^. Research is also expanding to explore the potential role of these sleep trackers in measuring sleep in comparison to traditional sleep diaries. However, the majority of these studies were conducted in healthy populations with small sample sizes, and we only identified one study in healthy population that compared Fitbit to a self-reported sleep measure ^30^. Similar to our findings, most studies using wearable sleep trackers identified moderate to high correlations between these devices and sleep diaries for TST ^31–33^. Additionally, both our study and the other study that used Fitbit showed less than a 20-minutes average difference in TST between Fitbit and CSD, indicating TST measured by Fitbit could be considered a reasonable estimate of TST obtained by CSD. However, unlike the other study, our study showed that Fitbit recorded longer, rather than shorter, TST relative to CSD. It is worth noting that both measures have limitations with regards to assessing TST: On one hand, wearable sleep trackers may identify low activity tasks, like still awakenings, as sleep, resulting in inaccurately longer sleep duration measurements for insomniacs compared to the healthy population ^34,35^. On the other hand, mental stress, physical discomfort or fatigue associated with insomnia or other health issues may cause people to suspect their sleep is inadequate and under-report sleep hours when using CSD ^36^.

With the exception of TST, the sleep metrics measured by Fitbit were statistically significantly different from those measured by CSD. Interestingly, consistent with another study ^32^, the magnitude of these differences was not substantial, suggesting that the sleep data obtained from Fitbit may be more reliable for long-term monitoring in a larger dataset than for short-term assessment in small populations or individuals. The difference was particularly pronounced for NWAK. One major way for Fitbit to generate data is through accelerometers and motion data ^31^, which may incorrectly distinguish between awakenings and sleep in some circumstances. For example, patients’ movements during sleep, such as adjusting the blankets or pillows, and other movements in bed, such as a partner rolling over or a pet changing positions in bed, can affect the readings of accelerometers ^37^, resulting in excessive awakening records. These limitations should be considered when interpreting sleep data obtained from Fitbit or other similar devices. Particularly for patients with insomnia, seeing the excessive numbers of awakening may contribute to increased psychological distress and appraisal of poor sleep and its impact. These should be further explored in future study to evaluate the potential harm of commercial sleep measures in clinical care settings.

Consistent with previous research, our results also found that sleep monitoring, whether through sleep diaries or device-generated, may not always reflect questionnaires of self-reported insomnia severity ^38,39^. Questionnaires such as ISI reflect how patients feel about their sleep experience and the impact of sleep disturbance on their daily life, while sleep monitoring through sleep diaries or wearable devices can provide detailed information about patients’ sleep habits, patterns, circadian rhythms, and other sleep related behaviors ^17,36^. Since questionnaires like ISI are usually quicker and easier for patients to complete, they can be the initial step for sleep assessment especially in daily clinical practice. Once sleep disturbance is identified, further sleep monitoring can help to distinguish actual sleep issues from all other comorbid discomforts that may contribute to perceived insomnia. Additionally, monitoring sleep can help identify specific sleep disorders or underlying causes of poor sleep such as behavioral issues. Thus, combining questionnaires and sleep monitoring enables clinicians to gain a comprehensive understanding of patients’ overall sleep condition ^36^, which is essential for informing optimal treatment decision making to improve sleep outcomes.

The value of wearable devices in cancer care and research is increasingly recognized ^40^. Our study indicates that Fitbit could provide limited but valuable insights into monitoring sleep. It could potentially replace CSD for assessing TST, particularly in large populations or if long follow-up periods are needed. Given that short sleep duration (< 6 hours) has been demonstrated as the most biologically severe phenotype of insomnia ^41,42^, Fitbit can be a valuable tool in detecting short sleepers who are at a higher risk of experiencing complications associated with insomnia. However, it is still important to use sleep trackers in conjunction with sleep diaries and other measures for accurate and comprehensive sleep assessments. It is also critical for patients to understand the limitations of sleep trackers. Some potential inaccurate Fitbit recordings, such as too many NWAK, may make people suspect their sleep condition is worse than it actually is, causing unnecessary anxiety ^43^. Additionally, patients with certain medical conditions, such as neuromuscular impairments, may not be able to use Fitbit because the tremors or unusual movements caused by these conditions may lead to inaccurate recordings due to the nature of accelerometers ^44^. Therefore, healthcare providers should inform patients about appropriate use and interpretation of Fitbit data, and they should be cautious in recommending it to patients with particular conditions.

Our study has some limitations that need to be considered. Firstly, the parent studies were conducted in cancer survivors with cognitive difficulties. Therefore, patients may have memory issues that compromise the accuracy of sleep diary reporting. However, participants with significant cognitive impairment were identified by the use of the Blessed Orientation-Memory-Concentration screening measure and not eligible for inclusion in these two studies. Secondly, both Fitbit and CSD sleep measurements were conducted at home, which makes it difficult for researchers to supervise patients for proper use. Nevertheless, our findings provide valuable insight into a real-world setting. Thirdly, we only tested one type of wearable device (Fitbit). Our results may not be applicable to other sleep tracking devices due to potential variations in their technology and algorithms. Furthermore, it is worth noting that the Fitbit version used in our study (Charge 4) is not the latest model, and there is currently no research available regarding potential differences in sleep outcomes between different Fitbit models. Lastly, most of our study participants were women. Study has shown that gender can impact patient-reported sleep outcomes and potentially influence the correspondence between subjective and objective sleep quality ^45^. Therefore, our findings may not be generalizable to men or the overall population.

Despite these limitations, this is the first study to investigate the relationship between commercial wearable device readings and self-reported sleep outcomes in a cancer population using data from randomized clinical trials. Our findings suggest that Fitbit can be a comparable alternative to CSD for measuring TST. However, it still should be used with caution for assessing awakenings or other sleep outcomes in cancer survivors. This study contributes novel insights into the potential applications of wearable devices in cancer care and research, highlighting the importance of proper use and data interpretation.

## Figures and Tables

**Table 1:** Participant characteristics (N = 62)

Characteristics	Mean (standard deviation) or Number (percent)
Mean age (SD), y	57.8 (11.7)
Gender	
Male	6 (9.7)
Female	56 (90.3)
Race	
White	42 (67.7)
Nonwhite^1^	20 (32.3)
Education	
Under college	8 (13.1)
College degree	22 (36.1)
Graduate or above	31 (50.8)
Employment	
Full time	30 (48.4)
Part time	9 (14.5)
Not currently employed	23 (37.1)
Cancer type	
Breast	44 (71.0)
Other^2^	18 (29.0)
Cancer stage	
0-	30 (48.4)
	17 (27.4)
	11 (17.7)
Unknown	4 (6.5)
Year since cancer diagnosis	5.9 (7.7)
Insomnia Severity Index	14.5 (5.7)

1 Other includes Black, Asian and more than one race.

2 Other cancer types included prostate, colorectal, gynecologic, and bladder.

**Table 2:** Comparison and correlation of sleep outcomes measured by Fitbit and CSD

	Mean (SD)		Median (range)		MSD (SD)^1^	P-value^2^	Correlation between Fitbit and CSD	
	Fitbit	CSD	Fitbit	CSD			Correlation coefficient (r)	P-value^3^
**TST**	394.6 (65.7)	380.0 (85.8)	412.0 (142.0–474.0)	392.0 (68.0–569.0)	14.6 (84.7)	0.38	0.41	0.001
**WASO**	67.7 (23.4)	39.0 (42.1)	64.0 (14.0–151.0)	21.0 (1.0–194.0)	28.7 (40.5)	<0.001	0.36	0.004
**NWAK**	19.6 (5.8)	2.9 (3.2)	22.0 (3.0–32.0)	2.0 (0.0–20.0)	16.7 (6.6)	<0.001	-0.04	0.75
**TIB**	463.4 (72.2)	487.9 (58.0)	483.5 (175.0–581.0)	487.5 (72.0–606.0)	−24.4 (71.5)	0.010	0.44	<0.001
**SE**	85.1 (4.6)	77.9 (15.4)	86.0 (72.0–95.0)	83.5 (17.0–96.0)	7.1 (14.4)	0.001	0.29	0.022

1 MSD, mean signed difference

2 P value of MSD (SD) using Wilcoxon Sum Ranks Test

3 P value of correlation between Fitbit and CSD

**Table 3:** Comparison of sleep outcomes measured by Fitbit and CSD between patients with no/mild insomnia and patients with clinical insomnia

	Fitbit Mean (SD)			CSD Mean (SD)		
	ISI ≤ 14	ISI > 14	P	ISI ≤ 14	ISI > 14	P
**TST**	393.0 (62.7)	396.2 (69.7)	0.75^b^	396.9 (79.9)	363.1 (89.3)	0.088
**WASO**	66.6 (22.5)	68.7 (24.5)	0.74^b^	30.5 (34.0)	47.5 (47.9)	0.090
**NWNK**	19.6 (5.9)	19.6 (5.9)	0.98^a^	2.0 (1.1)	3.8 (4.2)	0.009^2^
**TIB**	461.1 (66.4)	465.8 (78.5)	0.62^b^	488.3 (57.2)	487.5 (59.7)	0.96^1^
**SE**	85.1 (4.6)	85.0 (4.6)	0.92^b^	81.4 (13.9)	74.5 (16.2)	0.029^2^

1 independent-samples t test

2 Mann-Whitney Test
